# Addressing the social determinants of health through health system strengthening and inter-sectoral convergence: the case of the Indian National Rural Health Mission

**DOI:** 10.3402/gha.v6i0.20135

**Published:** 2013-03-01

**Authors:** Amit Mohan Prasad, Gautam Chakraborty, Sajjan Singh Yadav, Salima Bhatia

**Affiliations:** 1 Ministry of Health and Family Welfare, Government of India, New Delhi, India; 2 Oil India Limited, New Delhi, India; 3 Health of the Urban Poor (HUP) Program, Population Foundation of India (PFI), New Delhi, India; 4 National Rural Health Mission, Ministry of Health and Family Welfare, Government of India, New Delhi, India; 5 East Delhi Municipal Corporation, Government of Delhi, New Delhi, India; 6 Consultant, Policy and Planning, National Rural Health Mission, Ministry of Health and Family Welfare, Government of India, New Delhi, India

**Keywords:** equity, social determinants of health, health systems, India, National Rural Health Mission

## Abstract

**Background:**

At the turn of the 21st century, India was plagued by significant rural–urban, inter-state and inter-district inequities in health. For example, in 2004, the infant mortality rate (IMR) was 24 points higher in rural areas compared to urban areas. To address these inequities, to strengthen the rural health system (a major determinant of health in itself) and to facilitate action on other determinants of health, India launched the National Rural Health Mission (NRHM) in April 2005.

**Methods:**

Under the NRHM, Rs. 666 billion (US$12.1 billion) was invested in rural areas from April 2005 to March 2012. There was also a substantially higher allocation for 18 high-focus states and 264 high-focus districts, identified on the basis of poor health and demographic indicators. Other determinants of health, especially nutrition and decentralized action, were addressed through mechanisms like State/District Health Missions, Village Health, Sanitation and Nutrition Committees, and Village Health and Nutrition Days.

**Results:**

Consequently, in bigger high-focus states, rural IMR fell by 15.6 points between 2004 and 2011, as compared to 9 points in urban areas. Similarly, the maternal mortality rate in high-focus states declined by 17.9% between 2004–2006 and 2007–2009 compared to 14.6% in other states.

**Conclusion:**

The article, on the basis of the above approaches employed under NRHM, proposes the NRHM model to ‘reduce health inequities and initiate action on SDH’.

Differences are unavoidable but inequities can never be justifiable or acceptable. India has a long history of adhering to the ideology of equity where policy decisions to promote equity such as ‘reservations for the disadvantaged’ have been an integral part of the country's strategy since independence^1^,^2^. These principles have also found their way into the health sector and promoting equity in heath has always been high on the agenda of the Indian government. This aspect has been given greater emphasis since 2005 under the National Rural Health Mission (NRMH).

In the early 2000s, three distinct types of inequities and differences plagued India, namely

1. Urban–rural differences
2. Interstate differences
3. Intrastate differences

These included differences in the availability of health resources (input), health service delivery, and health outcome indicators.

## Urban–rural differences

**Graph 1:**
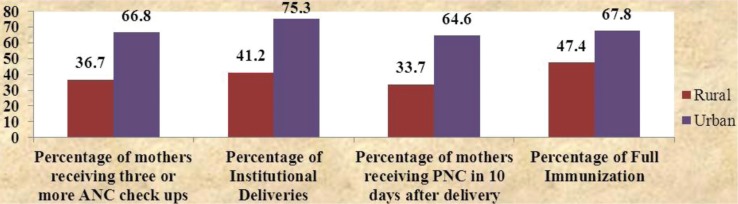
Difference in service delivery parameters between rural and urban areas (2).

In 2004, the infant mortality rate (IMR) in India was 24 points higher in rural areas (64/1,000 live births) as compared to urban areas (40/1,000 live births) (1). Similarly, there was a significant difference in health service delivery parameters. The percentage of mothers receiving three or more antenatal checkups varied from 36.7% in rural areas to 66.8% in urban areas (a difference of 30 points). The percentage of institutional deliveries varied from 41.2% in rural areas to 75.3% in urban areas (difference of 34 points). Similarly, there was a difference of 31 points between the percentage of mothers receiving postnatal checkups within 10 days after delivery and a difference of 20 points between the percentages of full immunization in rural and urban areas (2) (refer Graph 1).

## Interstate differences

While urban–rural differences were a stark reality, interstate differences were an equally significant cause for concern. In India, most northern and eastern states have historically fared poorer than the southern and western states, and eight of these socioeconomically backward states have been termed as Empowered Action Group (EAG)^3^ states by the Government of India. In 2004–2006, while EAG states struggled with a maternal mortality rate (MMR) of 375 (3), southern states were way ahead with less than half this figure (MMR-149). With regard to service delivery parameters in rural areas, states ranged from 99.5% institutional deliveries in Kerala to 10.2% institutional deliveries in Chhattisgarh (2). Similarly, the percentage of full immunization in rural areas ranged from 90.7% in Goa to 17.6% in Bihar (2).

**Graph 2:**
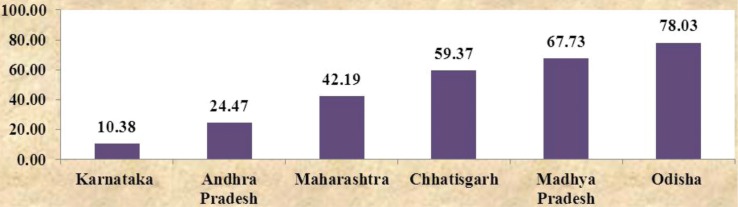
Shortfall in number of staff nurses across states (%) (4).

Unsatisfactory outputs are due to insufficient inputs. Interstate differences in outcome and output indicators were a result of deploying less than the required inputs. For example, the shortfall of nurses varied across states with just 10% in Karnataka to almost 80% in Odisha (4) (refer to Graph 2). It is thus evident that there were interstate differences in outcome indicators such as IMR/MMR, service delivery (output) indicators, such as institutional deliveries and full immunization, as well as input indicators, such as the shortfall of staff nurses against requirement.

## Intrastate differences

**Graph 3:**
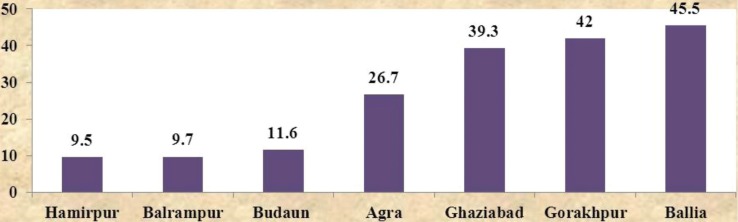
Percentage of pregnant women that received three or more antenatal checkups across districts in Uttar Pradesh (5).

However, it is essential to recognize at this point that while interstate differences were paramount, intrastate differences were also equally prevalent. In Uttar Pradesh, the percentage of women that received three or more antenatal checkups ranged from 45.5% in the district of Ballia to 9.5% in the district of Hamirpur (5) (refer Graph 3).

Recognizing the fact that the functioning of the health system has a considerable bearing on the health of the disadvantaged and vulnerable sections of the population, India launched the NRHM on the 12 April 2005 to provide accessible, affordable, and quality health care to the rural population, especially the vulnerable sections of the country (6).

In describing the NRHM, this article has a three-fold purpose. First and foremost, it aims to document the strategies employed under NRHM to achieve the goals of equity and convergence, and the first section of the article titled ‘Strategies to Achieve the Goals of Equity and Convergence’ provides a comprehensive overview of these approaches, against a backdrop of the recommendations made by the Commission on the social determinants of health (SDH), 2008.

Second, this article aims to document the impact of the mission in reducing inequities (i.e. in bridging the urban–rural and interstate divide) and fostering convergence, in the section on ‘Results and Discussion: Impact of the NRHM’. The parameters used for demonstrating the impact of the mission in reducing inequities are:

1. Improvement in health service delivery and health system strengthening in rural areas
2. Reduction in mortality in rural areas as compared to urban areas
3. Reduction in mortality in high-focus states as compared to other states
4. Reduction in out-of-pocket expenditures on health in rural areas as compared to urban areas.

Third, in the section on ‘Conclusion and Recommendations’, the article, on the basis of the above approaches employed under NRHM, proposes the NRHM model to ‘reduce health inequities and initiate action on SDH’, which may be employed by agencies working toward the above goals.

The basic data sources that have been used in this manuscript are the:

1. Sample Registration System (SRS) bulletins released by the Registrar General of India
2. Reports of the Coverage Evaluation Surveys (CES) by UNICEF
3. Bulletins on Rural Health Statistics (RHS), State-Wise Progress reports, and Common Review Mission reports released by the Ministry of Health and Family Welfare.

## Strategies to achieve the goals of equity and convergence

The Commission on SDH, set up by the World Health Organization to respond to the concern about widening inequities worldwide, has in its report called for action on SDH by improving daily living conditions; tackling the inequitable distribution of power, money, and resources; measuring and understanding the problem; and assessing the impact of action (7).

The Commission has further recommended improving daily living conditions by measures such as ‘promoting equity from the start’; aiming for ‘universal health coverage’; ‘building health care systems based on principles of equity, disease prevention, and health promotion’ among others. It has called for action on tackling the inequitable distribution of power, money, and resources by placing responsibility for health and health equity at the highest level of government; fair financing, strengthening public finance and fairly allocating government resources; promoting gender equity; promoting political empowerment and good governance. Measuring and understanding the problem and assessing the impact of action by monitoring, research, and training is the third important parameter identified for action on SDH (6).

The Commission has further rightly pointed out that the health system in itself is a significant determinant of health equity. To tackle health inequities, it is necessary for health systems not only to improve the services available but also to address the social determinants of health across many sectors (8).

In the Indian context, equity would mean reducing the urban–rural, interstate, and interdistrict divide by focusing on the rural areas, backward states and districts. It would also mean focusing on the scheduled caste and scheduled tribe populations and women and children, as these constitute the vulnerable populations.

The NRMH, launched in 2005 to provide health care to rural India, aimed at reducing the urban–rural divide and promoting action on SDH. The framework for implementation of the NRMH clearly stated that the mission would provide an opportunity for promoting equity and social justice. Developing a framework for promoting intersectoral convergence for promotive and preventive health care has been another important focus area.

**Fig. 1:**
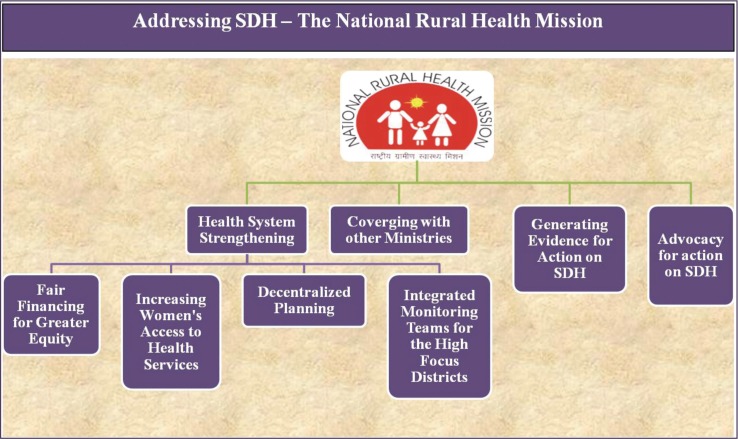
Strategies employed under NRHM to address social determinants of health.

Thus, the NRHM, though not designed in accordance to the recommendations of the Commission on SDH (released in 2008), has in essence been working largely parallel to the above mandates through strategies (Fig. 1), such as:

1. ‘Strengthening the health system’ in the rural areas of the country using the principles of ‘Fair Financing for Greater Equity’, ‘Increasing Women's Access to Health Services’, and ‘Decentralized Planning to Enable States to Address Local Priorities’
2. ‘Converging with the other Ministries’ to address key determinants such as early child development, nutrition, and sanitation
3. Generating evidence for action
4. Advocacy for action on SDH.

### Addressing social determinants of health through ‘health system strengthening’

NRHM aims to improve the daily living conditions and tackle the inequitable distribution of power, money, and resources by strengthening the health system through a three-pronged strategy by promoting ‘Fair Financing for Greater Equity’, ‘Decentralized Planning to Enable States to Address Local Priorities’, and promoting gender equity by ‘Increasing Women's Access to Health Services’.

#### Strengthening the health system in the rural areas of the country using the principle of ‘fair financing for greater equity’

As per the seventh schedule of the Constitution of India, public health and sanitation; hospitals and dispensaries are a part of the state list and are thus primarily the responsibility of the state governments (9). The delivery of health services to the people of the country and provision of the resources required to do so is a mandate of the state governments. However, as the rural areas across the country were seen to have worse health parameters than the urban areas, dedicated investments to the tune of Rs. 665.61 billion (US $12.13 billion) were made by the central government in the rural areas of the country by way of NRHM (10). NRHM also mandated a higher allocation to the 18 high-focus states with weak public health indicators and/or weak health infrastructure (which included the eight EAG states, eight northeastern states, Jammu and Kashmir, and Himachal Pradesh) (11). Moreover, it stipulated that states ensured higher allocation to 264 high-focus districts which include districts with poor health parameters, are populated by scheduled caste and scheduled tribe population, and are left wing extremism-affected, giving a weightage of 1.3 to the high-focus districts and 1.0 to the others (12).

#### Decentralized planning, enabling states to address their priorities – the corner stone of NRHM

As against the principle ‘One size fits all’, the foundation of NRHM is essentially based on the ideology of ‘decentralized planning based on local priorities’. The program mandates the preparation of state, district, and village action plans. Every year state government submits an annual State Program Implementation Plan (PIP), popularly known as the state PIP. While the NRHM division on an annual basis provides a framework for preparation of the action plan for the year, the scope for flexibility is immense. The broad strategies include support for infrastructure development, support for engagement of contractual human resources, including program management staff and addressing their training needs, support for referral transport, procurement of equipment/drugs, including strengthening supply chain management, support for mobile medical units, Information, Education and Communication/Behavior Change Communication (IEC/BCC), introduction of health volunteers called accredited social health activists (ASHAs) in villages, formation of Village Health and Sanitation Committees, community monitoring, provision of corpus grants to facilities (in the form of untied funds and annual maintenance grants), and research studies (12).

However, within the framework of these broad strategies, the state government based on local needs and priorities proposes the specific implementation strategy. For example, the type and number of contractual staff proposed by states for program management units varies considerably across states. Madhya Pradesh needs 50 contractual staff under its State Program Management Unit (SPMU), while Andhra Pradesh requires just 11. While each SPMU requires staff for 20–25 types of functional aspects, such as finance, legal functions, and IEC/BCC, taken together there are 64 different functional aspects across states (13). Thus, the number and type of staff proposed and approved vary considerably across states. Such flexibility is available for each of the above broad strategies.

Moreover, the Mission Flexible Pool component of the plan allows for proposal of funds for state-specific innovations, such as Sickle Cell Anemia Program, Mobile Mammography Vans, Tobacco Control Program, and much more. This phenomenal flexibility allows states to propose interventions addressing their local needs and priorities.

#### Increasing women's access to health services and focusing on gender equity

**Graph 4:**
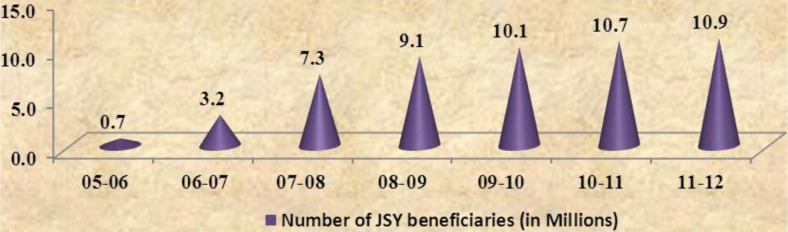
Increase in number of JSY beneficiaries in India from 2005–2006 to 2011–2012 (in millions) (10).

Increasing women's access to health services through the introduction of the Janani Suraksha Yojana (JSY) has been one of the prime objectives of the NRHM. JSY is a safe motherhood intervention under NRHM, implemented with the objective of reducing maternal and neonatal mortality by promoting institutional delivery among poor pregnant women. JSY integrates cash assistance with delivery and post-delivery care. The number of beneficiaries under the scheme has been steadily increasing since 2005–2006 and almost 5.40 crore women have benefitted from the scheme to date (6, 10) (refer Graph 4). Ministry has recently launched the Janani Shishu Suraksha Karyakaram with an aim to provide free to-and-from transport, free drugs, free diagnostics, free blood, and free diet to pregnant women and sick new-borns (14). The scheme has been launched in all states and UTs and is the first step toward a right-based approach to health services for women and children and is a significant step toward increasing women's access to health services.

NRHM has also renewed the focus on implementation of the Pre-conception and Pre-natal Diagnostic Techniques (Prohibition of Sex Selection) Act, 1994 (PC and PNDT Act) to check female feticides and address gender inequities (15).

### ‘Converging with other ministries’ to address key determinants such as ‘early child development’ and ‘sanitation’

Institutional mechanisms made mandatory from the central level such as the Mission Steering Group and Empowered Program Committee of NRHM, State and District Health Missions, Village Health and Nutrition Days, Village Health, Sanitation and Nutrition Committees, and Joint Mother and Child Protection Cards have been active measures for convergence with the other ministries.

At the national level, the NRHM functions under the overall guidance of the Mission Steering Group (MSG), which lays down the policies and programs for NRHM. The MSG fosters convergence at the policy level as Union Minister of Health and Family Welfare, Deputy Chairman Planning Commission, Minister of Rural Development, Minister of Panchayati Raj, Minister of Human Resource Development, and the secretaries of these ministries are part of the committee (16). The executive committee of the NRHM is called the Empowered Program Committee (EPC) and secretaries of all of the abovementioned ministries are members of the EPC (17). Structures such as the MSG have also been set up at the state and district levels in the form of State and District Health Missions. These committees provide an institutional platform for promotion of policy level convergence (18).

Village Health, Sanitation and Nutrition Committees (VHSNCs) have been set up at the village/gram Panchayat level under the leadership of the Sarpanch (village leader) and the health worker in the village for carrying out activities leading to improvements in health, sanitation, and nutrition of the villagers (19, 20). Each month, the workers of Ministry of Women and Child Development and the Ministry of Health and Family Welfare (21) jointly hold a Village Health and Nutrition Day (VHND) in the village. VHSNCs and Village Health and Nutrition Days provide an institutional platform for fostering convergence and addressing the social determinants of health at ground level.

Apart from these, utilizing the flexibility provided under NRHM, states have devised their own initiatives for action on SDH (22–24).

### Integrated monitoring teams for the high-focus districts

Special teams have been constituted by the Ministry of Health and Family Welfare to monitor progress in the identified high-focus districts of the country, vide order No. 244/DC (CH and I)/2011 dated 6 April 2011. These teams, consisting of government officials and technical consultants, visit the districts and monitor progress viz-a-viz the action plans for the year. The teams hold meetings with the district officials, including the District Magistrates to give feedback and suggestions on improving the implementation of the programs. Integrated monitoring teams, constituted for monitoring high-focus districts, is thus a recent strategy employed by the ministry to ensure focused attention in backward areas and bridge the interdistrict divide.

### Measurement and evidence for action on social determinants of health

Although a good number of surveys are conducted in India on a regular basis, data on interdistrict variability in health outcomes such as IMR and MMR, which was essential to further ensure the direction of investments, was not available through the existing surveys. Thus, realizing the need to prepare a comprehensive district health profile on key parameters based on a community set-up, the Annual Health Survey has been designed and introduced under NRHM to yield benchmarks of core vital and health indicators at the district level on fertility and mortality; prevalence of disabilities, injuries, acute and chronic illnesses and access to maternal health, child health, and family planning services (25). By virtue of being a panel survey, it has the unique ability to map the rate of change in these indicators on a yearly basis, making it a valuable tool for assessing the impact of action and for providing an evidence base for further action on SDH.

### Advocacy for action on social determinants of health

Ministry of Health and Family Welfare, Government of India, held a one-day National level Consultation on Social Determinants of Health on 27 February 2012, at New Delhi, to build a sustainable national movement for action on health equity and social determinants, linking governments, international organizations, research institutions, civil society, and communities. The ministry also released a book entitled ‘Unite for Health-Addressing the Social Determinants’ for insights into the concept of social determinants of health in Indian sociopolitical context and to motivate action on SDH (26). The discussions during this conference highlighted the need for institutionalizing structures for health impact assessment and the need for greater convergent action. The conference was in essence, the first step toward sensitizing stakeholders toward the need for action on SDH.

## Results and discussion: impact of the National Rural Health Mission

The strategies employed under NRHM have led to an improvement in availability of health services/health system strengthening, improvement in health service delivery, and have also had a significant impact on health outcomes in the rural areas, the high-focus states, and the high-focus districts in the country. Moreover, out-of-pocket expenditures on health in rural areas have shown a significant decline indicating that NRHM has also had a positive impact on reducing out-of-pocket expenditures. NRHM has also fostered action on other determinants of health such as nutrition and sanitation

### Improvement in availability of health services/health system strengthening

Under NRHM, contractual human resources have been employed in the rural areas and the facilities catering to the rural areas of the country. This has led to the addition of over 200,000 health providers in these areas, including Auxiliary Nurse Midwives, staff nurses, paramedics, doctors, AYUSH doctors (doctors of alternative medicine), and specialists. Apart from these, over 850,000 ASHAs (health volunteers introduced under NRHM at the village level) have been engaged to act as an interface between community and health care facilities (10).

Similarly, first referral units have been operationalized in these areas and there has been a 141% increase in the total number of first referral units (FRUs) as compared to the baseline year of 2005. It is has also been observed that high-focus states have shown a 625% increase (from 37 to 704) in the number of FRUs in comparison to non-high-focus states which have shown an increase of 85% (from 798 to 1,473) (27).

Construction/upgradation of over 46,000 health facilities; the addition of over 2,000 mobile medical units, over 14,000 ambulances, and emergency response vehicles; support for increasing availability of drugs, equipment, and diagnostic facilities, among many other initiatives, have also led to an increase in the availability of health services in rural areas in the country (10). Community participation and involvement of nongovernmental organizations has been fostered through community monitoring, public–private partnerships, and involvement of Panchayati Raj Institutions through VHSNCs and VHNDs.

There has been a significant increase in the number of pregnant women receiving three or more antenatal checkups in rural areas from 36.7% in 2005 to 63.3% in 2009 as per Coverage Evaluation Survey (CES) by UNICEF (2, 28). Moreover, many high-focus states have shown a better increase (Assam [40], Chhattisgarh [40], Himachal Pradesh [36], and Jammu and Kashmir [33]) as compared to the all-India average of 27 points as well as some non-high-focus states, namely Karnataka [17] and Andhra Pradesh [12].

Similarly, with regard to institutional delivery, high-focus states, such as Madhya Pradesh [46], Uttar Pradesh [49], Rajasthan [37], Assam [36], Odisha [35], Chhattisgarh [30], and Bihar [30], have all shown a better increase as compared to the national average of 28 points and some non-high-focus states, such as Haryana [16] and Gujarat [10] (2, 28). While it cannot be denied that non-high-focus states had good indicators to begin with and the magnitude of improvement seen in high-focus states was not feasible in non-high-focus states, it would be a folly to disregard the improvement in high-focus states based on this fact alone.

Most importantly, as pointed out in the report of the Third Common Review Mission of NRHM, the shift in load from secondary levels to primary is evident in several states and facilities. While there is a sustained increase in institutional deliveries against estimated deliveries in the state, in many districts there is a decrease in the number of deliveries at the district hospital, thus relieving them of the overcrowding and load.

### Improvement in health outcomes

In 2004, the IMR in India was 24 points higher in rural areas as compared to urban whereas per the latest SRS data, there is now a 19 point difference between the IMR in urban and rural areas. The average decline in rural IMR between 2004 and 2011 in large high-focus states is 15.6 points, whereas the average decline in urban IMR in these states during the same period is just 9 points (1, 29) (refer Table 1).

**Table 1:** Decline in rural and urban IMR across large high-focus states between 2004 and 2011

Names of large high-focus states	Decline in rural IMR between 2004 and 2011	Decline in urban IMR between 2004 and 2011
Bihar	18	13
Chhattisgarh	12	11
Himachal Pradesh	15	−5
Jammu and Kashmir	8	9
Jharkhand	10	6
Madhya Pradesh	21	17
Odisha	22	18
Rajasthan	17	10
Uttar Pradesh	15	12
Uttaranchal	18	−1
Average decline in large high-focus states	15.6	9

From Refs. 1 and 29.

**Graph 5:**
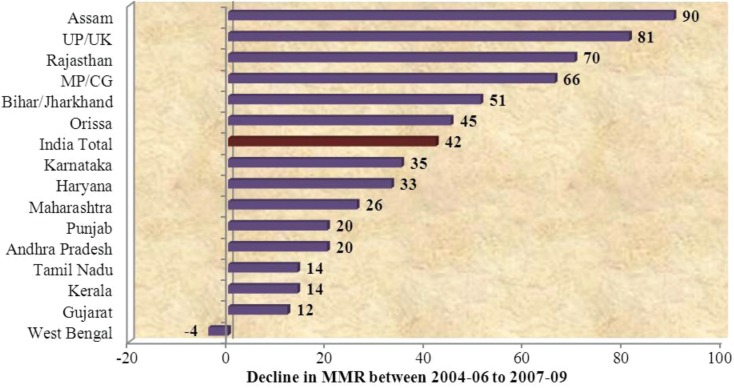
Decline in MMR between 2004–2006 and 2007–2009 (3, 30).

Data from SRS 2011 shows that in 2010–2011 most large high-focus states (7 out of 10) have shown a decline in IMR, which is better or equal to the national average of 3 points. Similarly, there is a greater decline in MMR of high-focus states between 2004–2006 and 2007–2009. Assam [90], Uttar Pradesh [81], Rajasthan [70], Madhya Pradesh [66], Bihar [51], and Odisha [45] have all shown a decline which is above the national average of 42 points (3, 30; refer Graph 5). MMR in high-focus states declined by 17.87% between 2004–2006 and 2007–2009, as compared to 14.57% in other states.

### Reducing out-of-pocket expenditures on health in rural areas

Between 2004–2005 and 2009–2010, at constant prices, total monthly per capita expenditure has increased by more than 7% in rural areas but per capita monthly heath expenditure has shown a decline of 7%. In comparison, at constant prices, the urban areas have seen a significant increase of 10.5% in out-of-pocket expenditure on health. Thus, from 2004–2005 to 2009–2010, the escalation of out-of-pocket expenses on health care has been reversed in rural areas while in urban areas, the upward spiral continues unabated. A major effort to make a difference in health care in rural areas over this period has been from the NRHM. The favorable trend indicates that NRHM has been able to make a positive impact on out-of-pocket expenditure on health in rural areas (31).

### Fostering convergence for action on other determinants of health

The MSG and EPC set up under NRHM have provided opportunities to bring about policy level convergence by activities such as incentivizing ASHAs, who are volunteers under the Ministry of Health and Family Welfare, for motivating households to construct and use a toilet which is a mandate of the Total Sanitation Campaign – a campaign run by the Ministry of Drinking Water and Sanitation for improving sanitation in rural areas. Similarly, a Joint Mother and Child Protection Card (Joint MCP Card) has been designed and circulated jointly by the Ministry of Women and Child Development and the Ministry of Health and Family Welfare to promote the continuum of care approach. This card traces the health of the woman during pregnancy, is used for monitoring the antenatal care and postnatal care provided to her, and also monitors the immunization and growth of the child for focus on early child development and equity from the start.

The VHSNCs set up at the village level under the NRHM have been known to carry out activities, such as sanitation drives, filling of pot holes, drives to control spread of vector-borne diseases, provision of nutritional support for malnourished children, purchase and installation of water purifiers at the Anganwadi centers (centers for the provision of Integrated Child Development Services) set up in the villages, which work effectively to address the determinants at the local level (20). Immunization of children; ante-natal checkup of mothers; growth monitoring of children; and counseling on nutrition, sanitation, and other topics related to the health of the mother and child is an important part of the Village Health and Nutrition Days held in the villages on a monthly basis.

Apart from the above pan-India mechanisms, some of the state-specific strategies for action on social determinants of health include the Nutritional Rehabilitation Centers set up in states, such as Madhya Pradesh and Bihar, to combat severe acute malnutrition (22); the Swasth Panchayat Yojana started by Chhattisgarh wherein Panchayats (local self-governments at village level) are ranked on 10 indicators for the awards and financial support, mobilizing them to take action on improving the determinants of health (23); and the School Health Program by Maharashtra wherein school health checkups are conducted in coordination with the Department of Education (24).

### Overall impact of the NRHM

Thus, it may be concluded that NRHM, launched to improve the health indicators of the rural areas, has in essence been a large step toward recognizing and taking corrective action on the health inequities within the country. It has clearly helped in reducing urban–rural and interstate inequities by improving the:

1. Availability of health resources
2. Health service delivery
3. Health outcomes in rural areas and high-focus states of the country.

Measuring the improvement in health outcomes in high-focus districts is slightly difficult due to the lack of district-wise data during the above periods. However, with the Annual Health Surveys being conducted under NRHM, disaggregated data across districts in high-focus states will be available from now on for assessing the impact of action.

The improvements in health outcomes are undoubtedly a result of dedicated investments in the rural areas, in the high-focus states, and in the high-focus districts, and NRHM has contributed greatly toward fair allocation between geographical regions and ethnic groups which, as is rightly pointed out by the Commission on SDH, is essential to promote equity. Strengthening health systems primarily through the tenet of fair financing for greater equity was thus one of the chief strategies employed by NRHM to reduce health inequities. ‘increasing women's access to health services’ and ‘decentralized planning to enable states to address local priorities’ are some of the other measures employed by NRHM to strengthen health systems.

*‘*Converging with the other ministries’ to address determinants such as early child development, nutrition, and sanitation; generating evidence for action and advocacy for action are the other measures employed by NRHM to address SDH. While it is difficult to gauge which of the above interventions have had maximum impact on the improvement in health outcomes, and the interpretation of this is beyond the scope of this article, the effect of the combination of the above strategies is undoubtedly evident.

## Conclusion and Recommendations

**Fig. 2:**
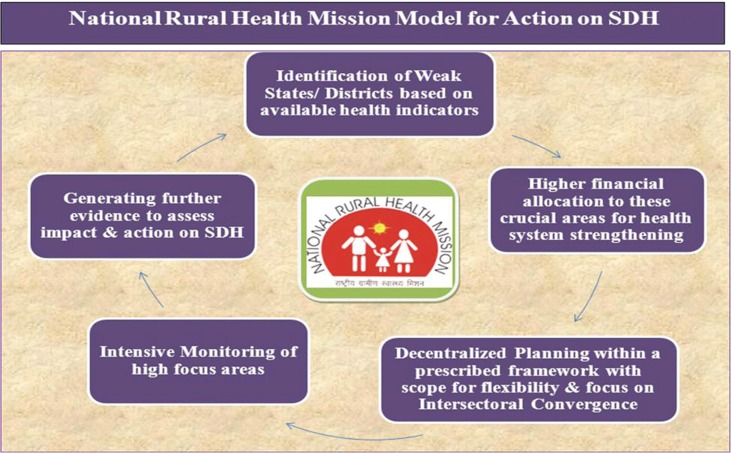
National Rural Health Mission model for action on SDH.

To conclude, it may be said that the NRHM has followed the following model to reduce health inequities and initiate action on SDH (Fig. 2):

1. The first step for initiation of action on SDH has been the identification of weak states/districts based on available health indicators.
2. Once identified, higher financial allocation has been ensured to high-focus states, and states have been encouraged to provide higher allocation to high-focus districts.
3. The weak states/districts have been encouraged to plan for themselves within a prescribed framework, with focus on promoting gender equity and institutional mechanisms for convergence such as State and District Health Missions, VHSNCs, Village Health and Nutrition Days, but with enough scope for flexibility and incorporation of local priorities and innovations, such as Nutritional Rehabilitation Centers, Swasth Panchayat Yojana, and School Health Programs. At the national level, convergence has been fostered with the help of the MSG and EPC.
4. Intensive monitoring of the high-focus areas followed by efforts to generate further evidence toward persistent inequities through surveys such as ‘Annual Health Surveys’ to ensure availability of disaggregated data for feedback into the cycle have been part of the strategies for action on SDH.

While these strategies have been successful in initiating action on social determinants of health, persistent inequities and varied socio-cultural and economic differences across India, further collaborations are needed between the existing programs of different ministries, such as the National Rural Employment Guarantee Scheme, the Total Sanitation Campaign, the Sarva Siksha Abhiyan, and other such schemes; health impact assessment cells at the national and state levels; strengthening systems for measuring health inequities (26), along with a need for more effective implementation of the currently designed policies.

However, policy makers and agencies working toward the goal of reducing inequities may consider the above model for action on SDH as NRHM has, without doubt, been instrumental in reducing the urban–rural and interstate inequities in India.

## Footnotes

1 The views expressed in this article are the authors’ personal opinions and do not in any way express the views of the Ministry of Health and Family Welfare/any other organization.

2 States with weak public health indicators and/or weak health infrastructure.

3 Assam, Bihar/Jharkhand, Madhya Pradesh/Chhattisgarh, Odisha, Rajasthan, Uttar Pradesh/Uttarakhand.
